# Bayesian Linear Regressions Applied to Fibromyalgia Syndrome for Understanding the Complexity of This Disorder

**DOI:** 10.3390/ijerph19084682

**Published:** 2022-04-13

**Authors:** Margarita I. Cigarán-Méndez, Oscar J. Pellicer-Valero, José D. Martín-Guerrero, Umut Varol, César Fernández-de-las-Peñas, Esperanza Navarro-Pardo, Juan A. Valera-Calero

**Affiliations:** 1 Department of Psychology, Universidad Rey Juan Carlos, 28922 Alcorcón, Spain; margarita.cigaran@urjc.es; 2 Intelligent Data Analysis Laboratory, Department of Electronic Engineering, ETSE (Engineering School), Universitat de València, 46100 Valencia, Spain; oscar.pellicer@uv.es (O.J.P.-V.); jose.d.martin@uv.es (J.D.M.-G.); 3 VALTRADOFI Research Group, Department of Physiotherapy, Faculty of Health, Camilo Jose Cela University, 28962 Villanueva de la Cañada, Spain; umut.varol@alumni.ie.edu (U.V.); javalera@ucjc.edu (J.A.V.-C.); 4 Department of Physical Therapy, Occupational Therapy, Rehabilitation and Physical Medicine, Universidad Rey Juan Carlos, 28922 Alcorcón, Spain; 5 Department of Developmental and Educational Psychology, Universitat de València, 46010 Valencia, Spain; esperanza.navarro@uv.es; 6 Department of Physiotherapy, Faculty of Health, Camilo Jose Cela University, 28692 Villanueva de la Cañada, Spain

**Keywords:** fibromyalgia syndrome, Bayesian Linear Regression, mathematical modeling, disability, statistical methods

## Abstract

A better understanding of the connection between factors associated with pain sensitivity and related disability in people with fibromyalgia syndrome may assist therapists in optimizing therapeutic programs. The current study applied mathematical modeling to analyze relationships between pain-related, psychological, psychophysical, health-related, and cognitive variables with sensitization symptom and related disability by using Bayesian Linear Regressions (BLR) in women with fibromyalgia syndrome (FMS). The novelty of the present work was to transfer a mathematical background to a complex pain condition with widespread symptoms. Demographic, clinical, psychological, psychophysical, health-related, cognitive, sensory-related, and related-disability variables were collected in 126 women with FMS. The first BLR model revealed that age, pain intensity at rest (mean-worst pain), years with pain (history of pain), and anxiety levels have significant correlations with the presence of sensitization-associated symptoms. The second BLR showed that lower health-related quality of life and higher pain intensity at rest (mean-worst pain) and pain intensity with daily activities were significantly correlated with related disability. These results support an application of mathematical modeling for identifying different interactions between a sensory (i.e., Central Sensitization Score) and a functional (i.e., Fibromyalgia Impact Questionnaire) aspect in women with FMS.

## 1. Introduction

Fibromyalgia syndrome (FMS) affects 0.2–6.6% of the population worldwide [1] and presents with a plethora of symptoms including widespread pain, fatigue, sleep disturbances, anxiety and/or depressive symptoms, and a decreased health-related quality of life and function [2]. This heterogeneity in clinical symptoms may be responsible for the difficulties with diagnosis in the clinical practice. Although signs and symptoms of FMS are generally well described, underlying mechanisms are poorly understood and currently FMS is explained in a biopsychosocial model where sensory, physical, and cognitive factors are interconnected. Fibromyalgia syndrome was described as a sensitivity syndrome [3], and more recently as a nociplastic condition [4].

The heterogeneity in clinical presentation observed in individuals with FMS has led to research examining the associations between psychological, psychophysical, physical, and cognitive variables with pain and related disability in women with FMS [5,6,7]. Identifying modifiable risk factors associated with pain sensitivity and related disability in people with FMS could assist therapists in determining more specific therapeutic programs for these patients. Previous studies used Pearson Product-Moment Correlations or linear regressions to determine the associations between the variables. These analyses exhibit some potential shortcomings. For instance, linear regression analyses usually use frequentist models, which consider different statistical assumptions to employ ordinary least square estimations [8].

Bayesian models are mathematical approaches that include information from a priori probability distribution which, when combined with the data’s likelihood function according to the Bayes theorem, can obtain the distribution of the variables [9]. The Bayesian model deals better than frequentist analysis with complex relationships since it learns the parameter distributions of the variable in a more plastic way [10]. The Bayes method is the pragmatic mathematical selection here since the models obtained are more precise and more restrictive than those obtained with a frequentist approach and are also able to directly calculate the predictive intervals [11].

In the current paper, we applied mathematical Bayesian modeling to identify associations between pain sensitivity symptoms, a common manifestation of sensitization, and related disability with clinical, psychological, psychophysical, health-related, and cognitive variables in women with FMS. The novelty of the current work was to potentially transfer the mathematical background to a complex pain condition with widespread and diffused symptomatology. Accordingly, the objective of our study was to identify potential associations between clinical, psychological, psychophysical, health-related, and cognitive variables with a sensory (i.e., sensitization pain-related symptoms) and a functional (i.e., pain-related disability) variable in a sample of women with FMS by using Bayesian Linear Regressions (BLR). We hypothesized that sensitization pain-related symptoms would be associated with psychological, psychological, and cognitive variables whereas pain-related disability would be more associated with clinical and health-related variables.

## 2. Materials and Methods

### 2.1. Participants

A consecutive sample of women with a medical diagnosis of FMS [12] voluntarily recruited from AFINSYFACRO Fibromyalgia Association, Madrid (Spain), by local announcements, were screened for eligibility criteria. Exclusion criteria included previous whiplash injury, previous surgery, any neuropathic condition (e.g., radiculopathy or myelopathy), any other underlying medical condition (e.g., tumor), or regular medication use affecting muscle tone or pain perception except symptomatic use of non-steroidal anti-inflammatory drugs (NSAIDs).

The Local Ethics Committee of Camilo José Cela University (UCJC 20-10-2020) and Universidad Rey Juan Carlos (URJC 08-30-2020) approved the study. Participants signed written informed consent prior to their inclusion in the study.

### 2.2. Output Variables: Pain Sensitivity and Related Disability

The output features (dependent variables) included in this Bayesian analysis were the Central Sensitization Inventory (CSI) for determining pain sensitivity (sensitization pain symptomatology) and the Fibromyalgia Impact Questionnaire (FIQ) for assessing pain-related disability. The CSI is a self-reported questionnaire evaluating 25 symptoms associated with sensitization with a 5-point Likert scale resulting in a score ranging from 0 to 100, where >40 points suggests the presence of sensitization [13]. The FIQ consists of 10 subscales focused on function during daily living activities, number of days feeling good or bad within the last week, whether FMS influences negatively with their working tasks, intensity of pain and fatigue, sleep quality, morning stiffness, and psychological features (i.e., anxiety and depression) [14]. Final scores range from 0 (no impact) to 100 (worst disability and severity attributable to FMS) [14]. In the current study we used the FIQ since it has been shown to exhibit good validity and psychometric properties [14].

### 2.3. Clinical Variables

A 10-point (0: no pain, 10: maximum pain) Numerical Pain Rate Scale [15] (NPRS) was used to evaluate pain intensity. Participants rated their mean score and their worst pain intensity at rest and their mean pain intensity with daily living activities on an NPRS. Since a high collinearity between the mean and worst pain intensity at rest is present, the mean value was calculated and used in the analyses.

### 2.4. Psychological Variables

The Hospital Anxiety and Depression Scale (HADS) was used to analyze whether the participants exhibited depressive or anxiety symptoms. This self-reported questionnaire includes seven items assessing anxiety levels (HADS-A) and seven items evaluating depressive levels (HADS-D). Each subscale provides a score ranging from 0 (absence of anxiety/depression) to 21 (greatest level of anxiety/depression) [16].

In addition, the Spanish version of the Pittsburgh Sleep Quality Index (PSQI) was used for evaluating sleep quality [17]. Twenty-four items aim to evaluate sleep quality within the last month by using questions regarding their usual bedtime, wake-up time, number of hours slept, and time needed to fall asleep. Each question is answered on a 4-point Likert scale (0 to 3), where greater scores suggest worse sleep quality [17].

### 2.5. Psycho-Physical Variables: Pressure Pain Thresholds

Pressure pain thresholds (PPTs) were used to determine widespread pain sensitivity. Pressure pain thresholds, e.g., the minimal amount of pressure where the patient first perceives pain, were measured bilaterally with an electronic algometer (Somedic AB©, Farsta, Sweden) over the mastoid process, upper trapezius muscle, elbow, hand, posterosuperior iliac spine, greater trochanter, knee, and tibialis anterior. Pressure was applied at a rate of approximately 30 kPa/s on each point. The mean of three trials on each point, with a resting period of 30 s between each, was calculated and used in the analysis. This testing procedure has been shown to have good reliability (ICC ≥ 0.88) in FMS [18]. Since no side-to-side differences were observed at any assessed point (independent Student *t*-tests), the mean of both sides was used in the BLR.

### 2.6. Cognitive Variables

We assessed pain hypervigilance behaviors by using the short-form of the Pain Vigilance and Awareness Questionnaire (PVAQ). The Spanish version of the PVAQ is a valid and reliable questionnaire (based on 9 items) used to identify whether patients constantly observe their pain perception [19].

Additionally, catastrophizing pain-related responses (e.g., constant worry, inability to avoid thoughts related with pain experience, heightened unpleasantness feelings, negative expectations about their pain or disease management, and inability to face pain) were evaluated using the Pain Catastrophizing Scale (PCS). This self-reported questionnaire consists of 13 items which are answered in a 5-point frequency Likert scale (where 0 is interpreted as “never” and 4 as “all the time”). Therefore, greater scores (up to 52 points) reflect greater catastrophizing behaviors. The validated Spanish version of the PCS was used in this study [20].

### 2.7. Health-Related Variables

Participants rated their self-perceived health status by using the Fibromyalgia Health Assessment Questionnaire (FHAQ) since this is a disease-specific questionnaire designed to assess function in this population. This questionnaire consists of 8 items (answered in a 4-point Likert scale). The mean of all items is calculated to obtain the final score, where 0 is associated with better function and health status and 3 with the worst function and perceived health [21].

In addition, quality of life was assessed by using the EuroQol-5D questionnaire [22]. This questionnaire evaluates mobility, self-care, daily activities independency, perceived pain, and depression/anxiety impact domains. Responses range from 1 (absence of problems) to 3 (severe problems). All responses are converted into a single index number from 0 (health state equivalent to death) to 1 (optimal health) according to standardized values [23].

### 2.8. Physical Variable

Physical condition was evaluated with the timed up and go (TUG) test since evidence suggests it is an easy-to-perform, valid, and reliable test providing valuable predictive information to identify individuals with high risk of falls, early disability onset, and risk of death. This test analyzes the time needed by the patient to: (1) stand up from an armchair without the help of the arms, (2) walk straight (at a comfortable and safe speed) to a line placed 3 m in front of the chair, (3) turn back, and (4) sit down again [24]. Additionally, the TUG has shown to be a reliable physical fitness test for assessing agility/dynamic balance in women with FMS [25].

### 2.9. Data Overview and Preprocessing

The data processing procedure conducted here was the same as previously used in a neuropathic pain condition such as carpal tunnel syndrome [26]. Briefly explained, missing values were imputed using k-nearest neighbors, and the standard score (or z-score) of all features was employed for fitting the BLR, which is a requirement and also helps with the interpretation of the results.

### 2.10. Bayesian Linear Regression (BLR)

The BLR is a translation of the traditional LR to the Bayesian framework, and is defined in Equation (1), where I is the intercept term of the BLR, θ is the vector of parameters (or weights), x are the input features, and $\hat{y}$ is the variable to predict, which has a mean I+x⋅θ (just like regular LR) and a standard deviation std. Weakly informative priors were employed.

(1) $$\begin{array}{rrr} I & ∼ & N\left(\mu =0.0,\sigma =10.0\right)\\ \theta & ∼ & N\left(\mu =0.0,\sigma =10.0\right)\\ std & ∼ & \mathrm{HalfCauchy}\left(\beta =10.0\right)\\ \hat{y} & ∼ & N\left(\mu =|I+x\cdot \theta ,|\sigma =std\right) \end{array}$$

By using the parameters from Equation (1), Equation (2) emerges, where P(θ,I|x,y) is the posterior probability distribution of the parameters θ and the intercept I of the BLR model (what we want to know), and it is proportional to the prior probability of θ and I, and the likelihood P(y|θ,I,x), both of which are known. Since P(θ,I|x,y) is known up to a normalization constant, Markov chain Monte Carlo (MCMC) methods can be employed to efficiently sample from it, thus obtaining a numerical estimation of this distribution.

(2) $$\begin{array}{rr} P(\theta ,I|x,y) & =\frac{P(y|\theta ,I,x)\cdot P(\theta ,I|x)}{P(y|x)}=\\ & =\frac{P(y|\theta ,I,x)\cdot P\left(\theta ,I\right)}{P(y|x)}\underset{\theta ,I}{\propto }\\ & \underset{\theta ,I}{\propto }P(y|\theta ,I,x)\cdot P\left(\theta ,I\right) \end{array}$$

### 2.11. Bayesian vs. Frequentist Statistics

In Bayesian statistics, the full probability distributions of I,θ are obtained by updating the priors based on the observed data x, y. Hence, the credibility of the predictions $\hat{y}$ can be easily assessed, since their distribution is known; similarly, the credible interval (the region where a given percentage, e.g., 95%, of the distribution falls) of the parameters of the model θ can also be immediately obtained. This is akin to, but different from the 95% confidence interval employed in the frequentist approach, which represents the interval where the true value for θ would fall 95% of the times when sampling data randomly from the population, making it a much less intuitive approximation.

## 3. Results

### 3.1. Participants

**Figure 1:**
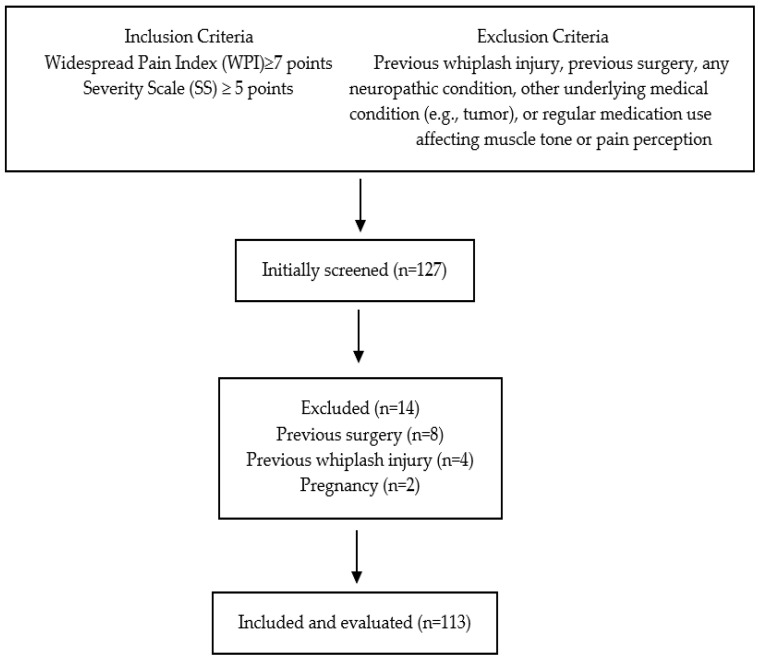
Flow diagram of patient recruitment.

From 127 women with FMS initially screened for inclusion and exclusion criteria, a total of 113 women (age: 52.8 ± 10.8 years) were included. Figure 1 shows the flow diagram with the inclusion and exclusion criteria. Table 1 summarizes all features (clinical, psychological, psychophysical, health-related, and cognitive data) employed for fitting the BLR. 

### 3.2. Bayesian Linear Regression

**Figure 2:**
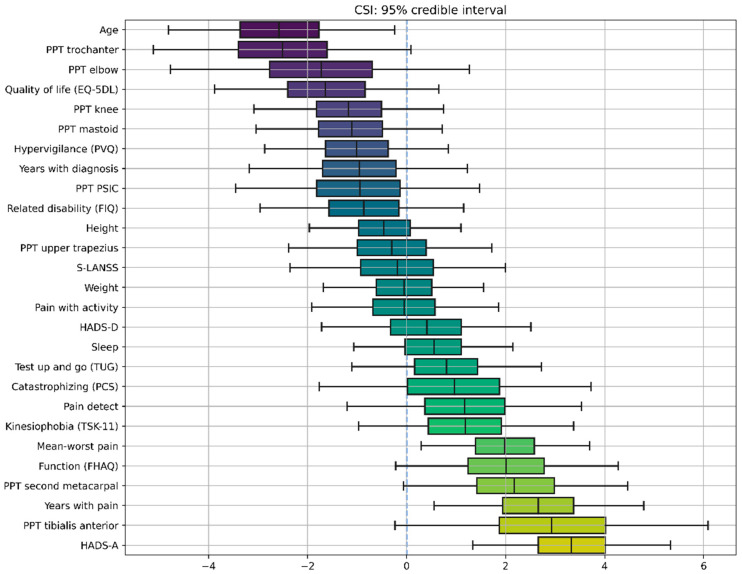
Credible intervals for all the parameters in the Bayesian Linear Regression (BLR) model for Central Sensitization Inventory (CSI). The X axis represents the values of the coefficients of the model, which are random variables, meaning that they do not have a specific value, but rather a “probabilistic value”, with some values being more probable than others. The negative sign (left side of the figure) means that the correlation with the CSI score is negative. The positive sign (right side of the figure) means that the correlation with the CSI score is positive. The magnitude, e.g., 3.5, indicates the relative strength of that correlation; it is relative in the sense that you can directly compare different coefficients because variables have been standardized. Accordingly, boxplots represent the distribution of a model coefficient, with whiskers enclosing its 95% credible interval. The 95% credible interval of a model coefficient is the range of values within which 95% of its probability falls.

**Figure 3:**
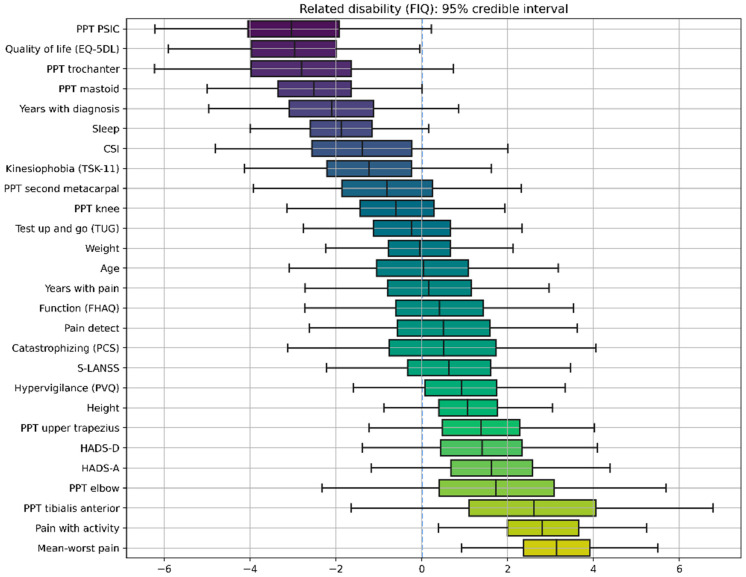
Credible intervals for all the parameters in the Bayesian Linear Regression (BLR) model for Fibromyalgia Impact Questionnaire (FIQ). The X axis represents the values of the coefficients of the model, which are random variables, meaning that they do not have a specific value, but rather a “probabilistic value”, with some values being more probable than others. The negative sign (left side of the figure) means that the correlation with the FIQ score is negative. The positive sign (right side of the figure) means that the correlation with the FIQ score is positive. The magnitude, e.g., 3.5, indicates the relative strength of that correlation; it is relative in the sense that you can directly compare different coefficients because variables have been standardized. Accordingly, boxplots represent the distribution of a model coefficient, with whiskers enclosing its 95% credible interval. The 95% credible interval of a model coefficient is the range of values within which 95% of its probability falls.

Two different BLR models, one for CSI score and the second for the FIQ, were obtained. Figure 2 and Figure 3 show the distribution of the variables learned by each model, with the caps at either side enclosing the 95% credible interval. If the credible interval does not cross the zero line, that parameter can be considered significant with a 95% credibility.

The first model revealed that age, pain intensity at rest (mean-worst pain), years with pain (history of pain), and anxiety levels (HADS-A) have significant correlations with the presence of sensitization-associated symptoms as assessed with CSI score (Figure 2). Additionally, PPTs over the tibialis and over the greater trochanter also exhibit strong correlations, although did not reach the 95% credibility threshold.

The second model showed that health-related quality of life (EuroQol-5D), pain intensity at rest (mean-worst pain), and pain intensity with daily activities have significant correlations with related disability (Figure 3), with PPT over the mastoid and sleep quality displaying strong, yet not significant, correlations.

## 4. Discussion

This is the first study applying Bayesian statistics for identifying the associations between pain-related, psychological, psychophysical, health-related, and cognitive variables with sensitization pain symptoms and related disability in women with FMS. The BLR showed that pain intensity (at rest or with activities) was intrinsically associated with a sensory-related and a functional-related variable. Further, anxiety levels (HADS-A) were also associated with sensitization pain symptoms (CSI score) whereas quality of life (EuroQol-5D) was associated with related disability/function (FIQ). Although some PPTs also showed associations, these did not reach the established credibility interval.

The first finding was that pain intensity construct (mean and worst intensity at rest and during daily living activities) was associated with either CSI (sensory) or FIQ (function) score. These findings support that the magnitude of the nociceptive peripheral input is a relevant factor to consider in FMS [27]. Additionally, the number of years with symptoms (i.e., longer chronicity) was also associated with a higher sensitization score. In such a scenario, a long-lasting duration (more time with pain) of the nociceptive input would also contribute to sensitization pain symptoms, i.e., temporal summation. This theory agrees with current knowledge showing that gray matter decreases are more pronounced with longer pain duration [28,29].

A second finding was that anxiety, but not depressive, levels were associated with sensitization pain symptoms. The association between mood disorders, depressive/anxiety levels, and the CSI score is not new in individuals with chronic pain since psychological factors have a significant impact on pain sensitivity [30,31]. It is possible that serotonergic and noradrenergic neuron dysfunction could affect psychological and somatic afferences leading to impaired descending inhibitory pathways [32].

The application of BLR also revealed potential, although not significant, associations between PPTs and sensitization symptoms. The presence of widespread pressure pain hyperalgesia is a feature of sensitization [33]. It is likely that, albeit not significant in our BLR, the presence of pressure pain hyperalgesia could have an effect on sensitization symptoms, although its contribution is questioned [34,35]. It is possible that PPTs represent a mechanism construct whereas the CSI represents a more clinical pain construct and, therefore, each variable assesses different aspects of the nociceptive pain spectrum. Nevertheless, it is important to consider that the CSI also involves psychological aspects of the pain experience, as it was expressed by a significant association with anxiety levels.

The second BLR showed that related disability (FIQ score) was associated with self-perceived health-related quality of life (EuroQol-5D). This is an expected finding since related disability is a functional outcome which would have a direct impact on self-perceived quality of life. In fact, pain-related fear is associated with pain-related disability [36], which could lead to a worse perceived health-related quality of life. As well as with PPTs, poor sleep quality displayed a potential, not significant correlation with related disability. Poor sleep quality could lead to lower energy during daily living activities, which would also contribute to worse self-perceived quality of life due to tiredness.

These results, based on mathematical models, have potential implications for clinical practice. First, the role of pain intensity would support the relevance of early treatment of pain in women with FMS with the aim to decrease sensitization pain symptomatology and related disability. In fact, several therapeutic strategies can be applied for decreasing pain intensity in FMS patients [37]. However, in this scenario, it is also important to consider that anxiety plays a relevant role in pain sensitization. Accordingly, psychological interventions would also be needed [38]. Our results suggest several interactions between pain-related variables and anxiety levels with sensory-related and related-disability variables. Accordingly, therapeutic approaches applied to women with FMS should consider all these interactions and be multimodal by including interventions targeting pain and disability (i.e., physical therapy approaches), mood disorders (i.e., cognitive behavior or stress management), and nociceptive pain processing (i.e., pharmacological or neuro-modulatory pain approaches) [39]. Nevertheless, it is important to consider that treatment interventions for FMS should be individualized according to the predominant symptom and mechanism [40].

Although the results from this study applying a mathematical model such as BLR are reliable, its limitations must be also considered. Firstly, BLR can be considered as a linear model and, as such, some complex relationships among the variables could be missed. Secondly, our results should be applied to just women with FMS. We do not currently know if the same associations would be found in men with FMS. Thirdly, we could originally consider the potential overlapping between psychological and cognitive variables with sensitization pain symptomatology; however, the lack of associations observed with BLR would support that the used questionnaires are able to cover different aspects of the complexity of FMS.

## 5. Conclusions

The current study has identified a potential interaction between the intensity/duration of pain and anxiety levels with self-reported sensitization pain symptoms. In addition, related disability was also associated with self-perceived health-related quality of life in women with FMS. Future studies investigating the clinical relevance of the current findings are still needed.

## Figures and Tables

**Table 1:** Clinical, psychological, psychophysical, health-related, and cognitive data of the sample (n = 113).

	Mean	SD	Min.	Max.
Age (years)	52.8	10.8	47.0	60.0
Height (cm)	160.25	36.4	156.0	169.0
Weight (kg)	72.45	16.8	57.0	110.0
Years with diagnosis	10.25	8.45	2.0	25.0
Mean-worst pain (NPRS, 0–10)	6.8	1.54	1.0	10.0
Pain with activity (NPRS, 0–10)	8.05	1.9	2.0	10.0
HADS-A (0–21)	11.5	3.8	1.0	20.0
HADS-D (0–21)	9.9	4.0	1.0	18.0
Sleep (PSQI, 0–21)	13.7	4.0	4.0	21.0
PPT upper trapezius (kPa)	134.7	56.2	50.45	273.8
PPT mastoid (kPa)	163.15	88.6	21.3	316.8
PPT elbow (kPa)	157.0	84.75	28.3	309.0
PPT second metacarpal (kPa)	126.7	56.55	15.5	294.0
PPT PSIC (kPa)	245.0	129.3	46.65	383.6
PPT trochanter (kPa)	271.1	119.4	74.5	421.8
PPT knee (kPa)	157.75	105.05	16.45	263.5
PPT tibialis anterior (kPa)	199.4	104.85	23.15	245.8
FIQ (0–100)	64.3	12.85	18.2	102.6
CSI Score (0–100)	70.25	11.95	36.0	99.0
Catastrophizing (PCS, 0–52)	22.6	12.35	0.0	47.0
Function (FHAQ, 0–3)	1.25	0.55	0.0	2.6
Hypervigilance (PVQ, 0–45)	27.25	8.1	8.0	47.0
Kinesiophobia (TSK-11)	25.0	7.55	11.0	43.0
Pain detect (0–38)	19.7	6.9	0.0	32.0
Quality of life (EQ-5DL, 0–1)	0.4	0.25	0.1	0.9
S-LANSS (0–24)	17.65	5.25	5.0	28.0
Test up and go (TUG, seg.)	12.35	4.7	4.45	29.7

NPRS: Numerical Pain Rate Scale; PPT: Pressure Pain Thresholds; S-LANSS: Self-reported version of the Leeds Assessment of Neuropathic Symptoms and Signs; CSI: Central Sensitization Inventory; HADS: Hospital Anxiety and Depression Scale (A: Anxiety, D: Depression); FIQ: Fibromyalgia Impact Questionnaire; FHAQ: Fibromyalgia Health Assessment Questionnaire; PCS: Pain Catastrophizing Scale; PVAQ: Pain Vigilance and Awareness Questionnaire.

## Data Availability

Not applicable.
